# Ganglioside sialylation modulates tau internalization and pathology spread

**DOI:** 10.1038/s41380-025-03394-2

**Published:** 2025-12-15

**Authors:** Shiying Li, Yuanyuan Chen, Tianling Song, Dong Liu, Ruozhen Wu, Xingyue Yang, Qian Wu, Leyi Lei, Xinyue Yu, Jing Zhang, Longfei Li, Yanli Jiang, Jianlan Gu, Jin Miao, Jin-hua Gu, Jianhua Shi, Feng Wu, Fei Liu, Dandan Chu

**Affiliations:** 1 Jiangsu Key Laboratory of Tissue Engineering and Neuroregeneration, Key Laboratory of Neuroregeneration of Ministry of Education, Co-innovation Center of Neuroregeneration, NMPA Key Laboratory for Research and Evaluation of Tissue Engineering Technology Products, Nantong University, Nantong, Jiangsu China; 2 School of Acupuncture-moxibustion, Tuina and Rehabilitation, Hunan University of Chinese Medicine, Changsha, Hunan China; 3 Department of Pharmacology, School of Pharmacy, Nantong University, Nantong, Jiangsu China; 4 Department of Biochemistry and Molecular Biology, School of Medicine, Nantong University, Nantong, Jiangsu China; 5 Laboratory of Animal Center, Nantong University, Nantong, Jiangsu China; 6 Department of Clinical Pharmacy, Affiliated Maternity and Child Health Care Hospital of Nantong University, Nantong University, Nantong, Jiangsu China; 7 Institute for Translational Neuroscience of the Second Affiliated Hospital of Nantong University, Center for Neural Developmental and Degenerative Research of Nantong University, Nantong, Jiangsu China; 8 Department of Neurochemistry, Inge Grundke-Iqbal Research Floor, New York State Institute for Basic Research in Developmental Disabilities, Staten Island, NY USA

**Keywords:** Neuroscience, Biochemistry, Cell biology, Molecular biology

## Abstract

Gangliosides serve as receptors for proteins, bacteria, and viruses, with sialylation at the termini of their glycan chains playing a crucial role in ligand recognition and endocytosis. The internalization of proteopathic tau aggregates by neurons is integral to the propagation of tau pathology in Alzheimer’s disease (AD). However, the influence of gangliosides and their sialylation modifications on the uptake of proteopathic tau aggregates and the subsequent impact on AD pathology remains unclear. This study investigates the roles of the four mammalian sialidases (Neu1-Neu4) in modulating tau aggregation in cellular models. Our findings demonstrate that Neu3 significantly inhibits tau aggregation induced by proteopathic tau derived from the brains of AD patients (AD P-tau). Overexpressing Neu3 or administering ganglioside GM1, which results from Neu3-catalyzed removal of one sialic acid from GD1a, in the mouse model decreases the GD1a/GM1 ratio in mouse brain, effectively blocks the spread of tau pathology and improves recognition in AD P-tau-injected mice. Both Neu3 and GM1 reduce the internalization of tau aggregates, while GD1a enhances tau uptake, showing a positive correlation with the level of internalized tau. Moreover, the internalization of tau mediated by GD1a dependent on low-density lipoprotein receptor-related protein 1 (LRP1) and compensates for heparin-inhibited tau uptake. In vitro assays demonstrate that GD1a exhibits a higher binding avidity for tau filaments than GM1. These findings indicate that GD1a may directly bind to tau aggregates via the sialic acid moiety, facilitating LRP1-mediated tau uptake. This study proposes a novel mechanism for tau internalization and posits that reducing ganglioside sialylation may be a promising strategy for hindering the spread of tau pathology in AD.

## Introduction

Alzheimer’s disease (AD) is the most prevalent neurodegenerative disorder leading to dementia in the elderly [1, 2]. It is characterized by two primary pathological features: extracellular amyloid plaques, resulting from the accumulation of β-amyloid (Aβ), and intracellular neurofibrillary tangles (NFTs) primarily composed of hyperphosphorylated tau proteins [3, 4]. NFT pathology appears a stronger correlation with cognitive decline than Aβ plaques in AD patients [5, 6]. Proteopathic tau serves as a template, facilitating the assembly of soluble dimers and oligomers that aggregate into insoluble paired helical filaments (PHFs) and NFTs, exhibiting prion-like properties [7]. Research indicates that toxic tau species from human brain propagate pathology in rodent brains through the prion-like mechanism, affecting anatomically connected regions in a manner similar to that observed in AD patients [8, 9]. Tau species sedimented from 27000 × g to 235000 × g from AD brain homogenate are termed proteopathic tau derived from AD brain (AD P-tau) leading to the most significant dissemination of pathology [10]. Therefore, inhibiting the intracellular transmission of prion-like tau species along connected neurons is essential for preventing the spread of tau pathology in AD brains.

Recent studies have illuminated the complex dynamics of tau protein release and uptake. Tau, released in either vesicular form or as free entities, is capable of being internalized by interconnected neurons in both anterograde and retrograde manners [11]. The mechanisms underpinning tau uptake are diverse and include pathways such as heparan sulfate proteoglycan (HSPG)-mediated endocytosis [12], clathrin-dependent endocytosis via low-density lipoprotein receptor-related protein 1 (LRP1) [13], direct penetration, vesicle transport, and endocytosis through tunneling nanotubes [14, 15]. Strategies aimed at targeting P-tau endocytosis and mitigating its propagation among neurons — such as the use of synthetic heparinoids [16] — have demonstrated efficacy in inhibiting tau uptake in cultured cell models. Additionally, heparin intervention is associated with significant delays in the age of clinical diagnosis for AD dementia [17], thus paving the way for the development of novel anti-AD therapeutic approaches.

Gangliosides are acidic glycosphingolipids characterized by the presence of sialic acids (N-acetylneuraminic acid, Neu5Ac), an acidic monosaccharide with a nine-carbon backbone at the termini of their glycan chains. Gangliosides are critical constituents of animal cell membranes and play significant modulatory roles in neuronal function and pathology [18]. Among the various gangliosides, the total content of the a-series disialo-ganglioside GD1a and monosialo-ganglioside GM1 in the adult brain is notable, exceeding 30% and making them some of the most abundant gangliosides present in the human brain [19]. Researches indicate that levels of GD1a and GM1 are typically reduced in AD brains [19]. Administration of GM1 has been associated with potential improvements in AD symptoms, although these improvements have only been observed in a limited cohort of patients [20, 21]. The interplay between gangliosides and AD pathology is complicated, encompassing both beneficial and detrimental interactions[18]. For instance, while interactions between the sugar moieties of GM1 and Aβ_42_ decrease the structural order of the Aβ_42_ dimer by diminishing its propensity to adopt a β-sheet configuration [22], GM1 also binds to γ-secretase and induces a conformational change in its structure. This interaction subsequently accelerates the cleavage of amyloid precursor protein (APP) by γ-secretase [23]. Despite these findings, the effects and mechanisms of gangliosides on the regulation of tau pathology in AD remain largely undefined.

The brain has the highest level of sialylation in the human body, with 65% of sialic acid bound to gangliosides [24]. Sialylation influences the biochemical properties of substrates, impacting various physiological processes including cell adhesion and recognition, as well as neuronal morphology and function [25]. Gangliosides also serve as receptors for bacteria and viruses, facilitating viral endocytosis by docking their sialic acid into viral proteins [26–29]. Given this, we questioned whether the negatively charged sialylation at the termini of gangliosides also mediate the internalization of proteopathic tau aggregates, like the role of negatively charged HSPGs?

Sialic acid is attached to either a galactose (Gal) or *N*-acetylgalactosamine (GalNAc) unit by sialyltransferases and is removed by sialidases/neuraminidases. This study investigated the four mammalian sialidases (Neu1-Neu4) in cell models, emphasizing that Neu3, which primarily targets gangliosides rather than glycoproteins [30, 31], significantly inhibited AD P-tau induced tau aggregation. Neu3 is a plasma-membrane-bound sialidase [32] that modifies the substrate disialo-ganglioside GD1a, resulting in the formation of monosialo-ganglioside GM1. We then concentrated on the effects of Neu3 and ganglioside GM1 on tau pathology and cognitive function in the AD mouse model. We also explored the uptake of tau aggregates regulated by Neu3, along with its substrate GD1a and product GM1. Additionally, we assessed the binding avidity of GD1a and GM1 for both tau monomers and filaments. Our research indicates a potential mechanism through which gangliosides may act as co-receptors for tau uptake, highlighting the role of ganglioside sialylation in tau pathology and paving the way for future tau-targeted therapeutic strategies in AD.

## Materials and methods

### Cell culture

All cell lines were obtained form ATCC (Washington, DC, NW, USA) and tested negative for mycoplasma contamination. The cells were cultured in Dulbecco’s Modified Eagle Medium (DMEM) supplemented with 10% fetal bovine serum (Thermo Fisher Scientific, Waltham, MA) at 37 °C in a 5% CO2 atmosphere. Plasmid transfections were carried out using X-tremeGENE™ HP DNA Transfection Reagent (MilliporeSigma, Bedford, MA, USA), while siRNA transfections were performed with Lipofectamine™ 2000 (Invitrogen, Carlsbad, CA, USA), following the manufacturer’s protocols.

The pCI/hemagglutinin (HA)-tau_151-391_ plasmids were prepared as previously described [33]. The plasmids pcDNA3.1 + /NEU1-C-(K)-DYK (OHu26827D), pcDNA3.1 + /NEU4-v2-C-(K)-DYK (OHu24025D), and pcDNA3.1 + /NEU3-C-(K)-DYK (OHu30315D)were obtained from GenScript, Nanjing, China, while pCMV3/NEU2-C-DDK (HG15845-CF) was acquired from Sino Biological, Beijing, China. Control transfections were conducted using empty vectors corresponding to the respective experimental conditions. siCon (5’-UUCUCCGAACGUGUCACGUTT-3’), siNeu3 (5’-GGUUACAGUAGAAUGUGAAGU-3’) were provided by Ibsbio, Shanghai, China. siLRP1 was acquired from Santa Cruz (Santa Cruz, CA, USA). GM1 (860065 P), GD1a (860055 P) and heparin (H3149) were purchased from MilliporeSigma.

AD P-tau was isolated from frozen autopsy cerebral cortex of AD patients, as detailed in previous studies [34]. The use of AD P-tau was exempted by the Institutional Review Board of New York State Institute for Basic Research in Developmental Disabilities on the grounds that “the research does not involve intervention or interaction with the individuals,” nor “is the information individually identifiable.” Prior to use, AD P-Tau was sonicated using Fisherbrand™ Model 505 Sonic Dismembrator (Fisher Scientific, Waltham, MA, USA) for 5 min (1 s on, 3 s off) at 80% power. To induce aggregation, AD P-tau was added to the culture at a final concentration of 6.6 μg/mL.

To separate RIPA**-**insoluble and soluble fractions [33, 35–37], cells were lysed in RIPA buffer containing 50 mM Tris-HCl, 150 mM NaCl, 0.1% SDS, 0.5% sodium deoxycholate, 1% Nonidet P-40, 50 mM NaF, 1 mM Na_3_VO_4_, and 10% protease inhibitor cocktail (04693132001, Roche, Basel Switzerland) for 20 min on ice. The lysate was then centrifuged at 130000 × g for 45 min at 4°C (Beckman, Indianapolis, IN). The resulting supernatant constituted the RIPA-soluble fraction, while the pellet was washed twice with RIPA buffer. The pellet was subsequently resuspended and sonicated for 10 min (1 s on, 3 s off) at 80% power, and this preparation was designated as the RIPA-insoluble fraction.

To obtain the SDS-soluble or urea-soluble fractions, the RIPA-insoluble pellet was resuspended in either SDS lysis buffer (1% SDS in 50 mM Tris, 150 mM NaCl pH 7.6) [38] or urea buffer (4 M urea, and 25 mM Tris-HCl, pH 7.6) [39, 40]. The mixture was then sonicated for 10 min (1 s on, 3 s off) at 80% power. Following centrifugation at 100,000 × g for 30 min at 22°C, the supernatants were collected and designated as the “SDS-soluble fraction” or “urea-soluble fraction”.

### Animals

B6;129-Tg(APPSwe,tauP301L)1Lfa *Psen1*^*tm1Mpm*^/Mmjax (3xTg-AD, #034830), B6129SF2/J (wildtype control for 3xTg-AD mice, #101045), B6;C3-Tg(Prnp-MAPT*P301S)PS19Vle/J (PS19, #008169) and littermate control mice were sourced from the Jackson Laboratory (New Harbor, ME, USA). The female 3xTg-AD [41, 42] and male PS19 mice [43] were used in this study. All mice were housed at a density of 4-5 individuals per cage, maintained on a 12-h light/dark cycle, and provided with food and water ad libitum. The animals were randomly allocated into experimental groups. Experimenters were blinded to the genotypes of the animals. All procedures involving animal care were ethically approved by the Administration Committee of Experimental Animals and adhered to the Institutional Animal Care Guidelines of Nantong University.

For stereotactic injection, mice were anesthetized using a 1.25% solution of tribromoethanol (Avertin) (MilliporeSigma). The coordinates for unilateral hippocampal injection were established as follows: -2.5 mm anterior/posterior, +2.0 mm medial/lateral relative to the bregma, and -1.8 mm dorsal/ventral from the dura mater. An injection volume of 2 μL of Adeno-associated virus (AAV, 5 × 10^12^ viral genome copy/mL) was used for this procedure. For intracerebral injection, the coordinates were -0.5 mm anterior/posterior, +1.0 mm medial/lateral to the bregma, and -2.5 mm dorsal/ventral from the dura mater, with an injection volume of 5 μL. The injection was performed at a rate of 0.5 μL/min, and the needle was retained in position for 5 min post-injection to minimize the risk of leakage. The dosage of AD P-tau administered was 0.55 μg/μL, with a total volume of 1 μL injected. GM1 was administered intraperitoneally at a dosage of 50 mg/kg [44] for 5 consecutive days, repeated once a month over a period of 5 months, to 20-month-old wild-type mice.

### AAV production and purification

AAV9-hSyn-Neu3-T2A-EGFP and AAV9-hSyn-EGFP were produced by Genechem, Shanghai, China. Briefly, the Neu3-T2A-EGFP or EGFP constructs were cloned into the CV235 plasmid, which facilitates the gene expression under the control of the human synapsin 1 (hSyn) promoter. Subsequently, these constructs were packaged into AAV9.

### Tau fibrillization and uptake assay

The recombinant tau_151-391_ protein was expressed and purified by Sangon Biotech (Shanghai, China). To prepare tau fibrils, 1 mg/mL tau_151-391_ protein was dissolved in PBS containing 1 mM dithiothreitol, 0.5 mg/mL heparin, and a 10% cocktail of proteinase inhibitors. The mixture was incubated at 37 °C without agitation for 24 h [35]. The fibrillized tau were then sonicated for 2.5 min (1 s on, 3 s off) at 50% amplitude prior to labeling. Tau fibrils were labeled with Alexa Fluor™ 555 Microscale Protein Labeling Kit (Thermo Fisher Scientific) according to the supplier’s instructions, referred to as TF555.

SH-SY5Y cells were plated at 80000 cells per well in an 8-well Lab-Tek II Chamber Glass Slide™ (Nunc, Rochester, NY, USA). Forty-eight hours post-transfection or 24 h after GM1 (80 μM), GD1a (80 μM), anti-LRP1 (5 µg/mL), or heparin (20 µg/mL) treatment, the cells were treated with TF555 (800 nM) for 2.5 h at 37 °C. For the co-treatment with siLRP1 and GD1a, the cells were first transfected with siLRP1. Twenty-four hours after transfection, the cells were treated with GD1a for an additional 24 h, followed by treatment with TF555. Following TF555 exposure, the cells were washed with PBS and trypsinized for 1.5 min to detach them from the plate. After a recovery period of 3 h of culturing, the cells were washed with PBS, fixed, and subsequently subjected to immunostaining. Each experiment was repeated 3-4 times, with a minimum of 10 images randomly captured for each repeat. The intensity of TF555 in the cells exhibiting the strongest internalization in each image was measured using Adobe Photoshop, and the average intensity was calculated. Finally, the average intensity was normalized to the control.

### Pull-down assay

The pull-down assay was conducted using the Pierce™ Classic Magnetic IP/Co-IP Kit (88804, Thermo Fisher Scientific). A concentration of 10 µg/mL of tau_151-391_ monomer or filament, combined with a 10% cocktail of proteinase inhibitors, was incubated with 6.4 µM of GD1a or GM1 for 2 h. Then, rabbit or mouse anti-Tau antibodies were added and incubated for an additional 2 h. Subsequently, Pierce™ Classic Magnetic beads were introduced and incubated for 1 h to precipitate tau protein. All procedures were performed at room temperature. The precipitates were then subjected to dot blot analysis to detect gangliosides. Rabbit IgG (A7058, Beyotime) was used as a control.

To assess the avidity of GD1a and GM1 for tau aggregates, 10 µg/mL tau_151-391_ filaments containing a 10% cocktail of proteinase inhibitors were incubated with concentrations of 0, 6.25 μM, 12.5 μM, 25 μM, 50 μM, 100 μM or 200 μM of GD1a or GM1 for 2 h at room temperature. Following ultracentrifugation at 130000 × g, the pellets were washed twice with TBS, resuspended in TBS, and sonicated for 5 min (1 s on, 3 s off) at 80% power before being subjected to dot blot analysis.

### Surface plasmon resonance (SPR)

The SPR assay was performed by Detaibio, Nanjing, China. Briefly, the target protein tau_151-391_ was diluted in 10 mM acetate (pH 4.5) to a concentration of 20 μg/mL. It was then applied to the surface of a CM5 chip (Cytiva, Chicago, IL, USA) at a flow rate of 10 μL/min for a duration of 420 s. Then the chip surface was blocked with 1 M ethanolamine (pH 8.5). The concentration gradients of GM1 included 15.6 μM, 31.2 μM, 62.5 μM, 125 μM, 250 μM, and 500 μM. For GD1a, the concentration gradients were 7.8 μM, 15.6 μM, 31.2 μM, 62.5 μM, 125 μM, and 250 μM. The flow rate was set to 30 μL/min, with a binding time of 120 s and a dissociation time of 360 s. The resulting signals were analyzed by fitting to either a 1:1 Langmuir binding model or a steady-state model using BIAcore T200 evaluation software.

*Additional details regarding the materials and methods can be found in the supplementary information*.

## Results

### Neu3 overexpression reduces AD P-tau-induced tau aggregation and decreases GD1a/GM1 ratio in cultured cells

**Fig. 1:**
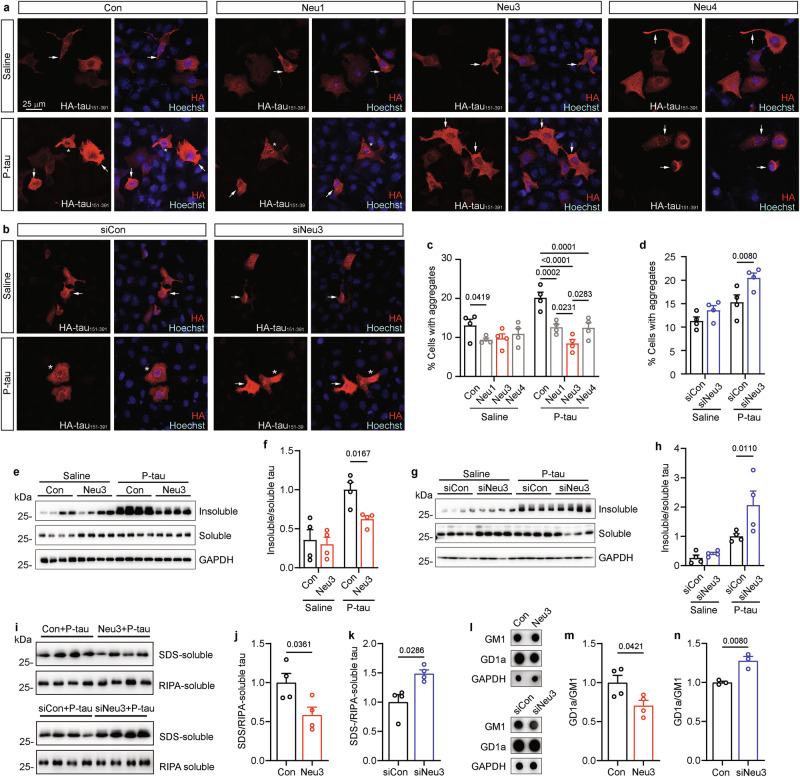
Neu3 inhibits AD P-tau-induced tau aggregation in cultured cells. **a** HeLa cells were co-transfected with the plasmid pCI/HA-tau_151-391_ and either pcDNA3.1/Neu1, pcDNA3.1/Neu3, or pcDNA3.1/Neu4. Tau aggregation was induced with AD P-tau, and cells were analyzed through immunofluorescence using anti-HA antibodies. Hoechst stains the nuclei. Arrow, cells with cytoplasmic aggregates. Asterisk, cells with nuclear aggregates. **b** HeLa cells were co-transfected with pCI/HA-tau_151-391_ and siNeu3, followed by AD P-tau induction for tau aggregation, analyzed similarly with anti-HA antibodies. **c** Quantification of HA-positive cells containing tau aggregates in **a** (n = 4) was analyzed by two-way analysis of variance (ANOVA), P-tau: *F*_1,24_ = 9.595, *P* = 0.0049; sialidases: *F*_3,24_ = 14.07, *P* < 0.0001; P-tau × sialidases interaction: *F*_3,24_ = 4.191, *P* = 0.0161. **d** Quantification from **b** (n = 4) was analyzed by two-way ANOVA, AD P-tau: *F*_1,12_ = 22.09, *P* = 0.0005; siNeu3: *F*_1,12_ = 10.47, *P* = 0.0071. **e** HEK293T c**e**lls were co-transfected with pCI/HA-tau_151-391_ and pcDNA3.1/Neu3, followed by AD P-tau induction for tau aggregation. The RIPA-insoluble and soluble fractions were examined by Western blots using anti-HA antibodies. GAPDH in RIPA-soluble fractions served as a loading control. **f** Quantification of the insoluble to soluble tau ratio in **e** (n = 4) was analyzed by two-way ANOVA, AD P-tau: *F*_1,12_ = 25.28, *P* = 0.0003; Neu3: *F*_1,12_ = 5.050, *P* = 0.0442. **g** HEK293T cells were co-transfected with pCI/HA-tau_151-391_ and siNeu3, followed by AD P-tau induction for tau aggregation. The RIPA-insoluble and soluble fractions were analyzed via Western blot using anti-HA antibodies. **h** Quantification of the ratio of insoluble to soluble tau in **g** (n = 4) was analyzed by two-way ANOVA, P-tau: *F*_1,12_ = 22.98, *P* = 0.0004; siNeu3: *F*_1,12_ = 5.807, *P* = 0.0329. **i** HEK293T cells were co-transfected with pCI/HA-tau_151-391_ and Neu3 or siNeu3, followed by AD P-tau induction for tau aggregation. The resulting SDS-soluble and RIPA-soluble fractions were analyzed via Western blotting using anti-HA antibodies. **j** Quantification of the ratio of SDS-soluble to RIPA-soluble tau in Neu3-expressed cells (n = 4) was analyzed by Student’s *t* test. k Quantification of the ratio of SDS-soluble to RIPA-soluble tau in siNeu3-treated cells (n = 4) was analyzed by Student’s *t* test. **l** HEK293T ce**l**ls were transfected with Neu3 or siNeu3, and the cell lysates were analyzed via dot blot analysis, with GAPDH serving as a loading control. **m** Quantification of GD1a/GM1 ratio in Neu3-expressed cells (n = 4) was analyzed by Student’s *t* test. **n** Quantification of GD1a/GM1 ratio in siNeu3-treated cells (n = 3) was analyzed by Student’s *t* test.

To explore the role of sialidases in tau aggregation seeded by proteopathic tau, human cervical cancer HeLa cells were co-transfected with pCI/HA-tau_151-391_ and plasmids for human sialidases Neu1 ~ Neu4. Six hours post-transfection, AD P-tau was introduced to the culture to induce aggregation. After 42 h, immunofluorescence using anti-HA antibodies was conducted to quantify tau aggregate-containing cells among HA-positive cells. Consistent with our previous findings [33, 35], HA-tau_151-391_ overexpression led to mild cytoplasmic aggregation in a subset of cells (~13%) (Fig. 1a, c). AD P-tau treatment further escalated aggregation in the nucleus and cytoplasm, elevating the aggregate-positive cell ratio to 20% (Fig. 1a, c). Excluding Neu2 (Supplementary Figure 1e), sialidases Neu1, Neu3, and Neu4 were found to efficiently reduce the AD P-tau-induced aggregation (Fig. 1a, c). Notably, Neu3 expression in HeLa cells markedly curbed tau aggregation most effectively, surpassing the impact of Neu1 and Neu4 (Fig. 1c). Next, HeLa cells were co-transfected with pCI/HA-tau_151-391_ and siNeu3, and followed by assessment of AD P-tau-induced aggregation. Immunofluorescence outcomes showed that siNeu3 significantly upregulated the fraction of cells exhibiting tau aggregates (Fig. 1b, d).

Next, pCI/HA-tau_151-391_ and pcDNA3.1/Neu3 plasmids were co-transfected into human embryonic kidney HEK293T cells, followed by AD P-tau addition to trigger tau aggregation. Forty-eight hours post-transfection, radio immunoprecipitation assay (RIPA) buffer-soluble and insoluble fractions were separated from the cell lysates and analyzed via Western blot using anti-HA antibodies [33, 45]. The addition of AD P-tau notably increased the proportion of RIPA- insoluble tau, whereas Neu3 overexpression (Supplementary Figure 1a-b) substantially reduced AD P-tau-induced insoluble tau aggregates (Fig. 1e, f). In contrast, co-transfecting pCI/HA-tau_151-391_ and siNeu3 (Supplementary Figure 1c-d) into HEK293T cells markedly raised the levels of RIPA-insoluble tau (Fig. 1g, h).

Subsequently, the RIPA-insoluble pellets were resuspended in either SDS buffer or urea buffer and subjected to ultracentrifugation to isolate and analyze the SDS-soluble or urea-soluble tau. Overexpression of Neu3 significantly decreased the levels of SDS-soluble tau, while siNEU3 markedly increased them (Fig. 1i-k). However, neither the overexpression nor knockdown of Neu3 had a substantial impact on urea-soluble tau (Supplementary Figure 1j-l). These results suggest that Neu3 plays a crucial role in inhibiting AD P-tau-induced SDS-soluble tau aggregation in cultured cells.

Since Neu3 is a sialidase that converts disialo-ganglioside GD1a to monosialo-ganglioside GM1, we employed dot blot analysis to investigate how its overexpression or knockdown influences the levels of GD1a and GM1. In HEK293T cells, the overexpression of Neu3 led to a significant reduction in GD1a levels without substantially affecting GM1 (Fig. 1l, Supplementary Figure 1f-g), resulting in a notable decrease in the GD1a/GM1 ratio (Fig. 1m). Conversely, the transfection of siNeu3 caused a significant decrease in GM1 while leaving GD1a levels unchanged (Fig. 1l, Supplementary Figure 1h-i), consequently increasing the GD1a/GM1 ratio (Fig. 1n). These findings imply that Neu3 plays a role in modulating the GD1a/GM1 ratio.

### Neu3 alleviates spatial memory defects and tau pathology in AD P-tau-injected 3xTg-AD mice

To investigate the in vivo function of Neu3 in an AD model, adeno-associated viruses expressing Neu3 (AAV-Neu3) were constructed under the control of the neuron-specific promoter synapsin 1. The viruses were injected into the lateral ventricle of 6-month-old female 3xTg-AD mice, followed by Morris water maze testing at 12 months of age (Supplementary Figure 2a). However, no significant changes in learning and memory were noted among the mice (Supplementary Figure 2a-e). We also administered AAV-Neu3 into the hippocampus of 6-month-old 3xTg-AD mice (Supplementary Figure 2f, h-k) and 4-month-old PS19 mice (Supplementary Figure 2g, 2l-o). Two months later, there were no significant changes in spatial learning and memory in either mouse model as assessed by the water maze (Supplementary Figure 2f-o), indicating the limited role of Neu3 in mitigating the cognitive impairments caused by overexpression of tau mutants.

**Fig. 2:**
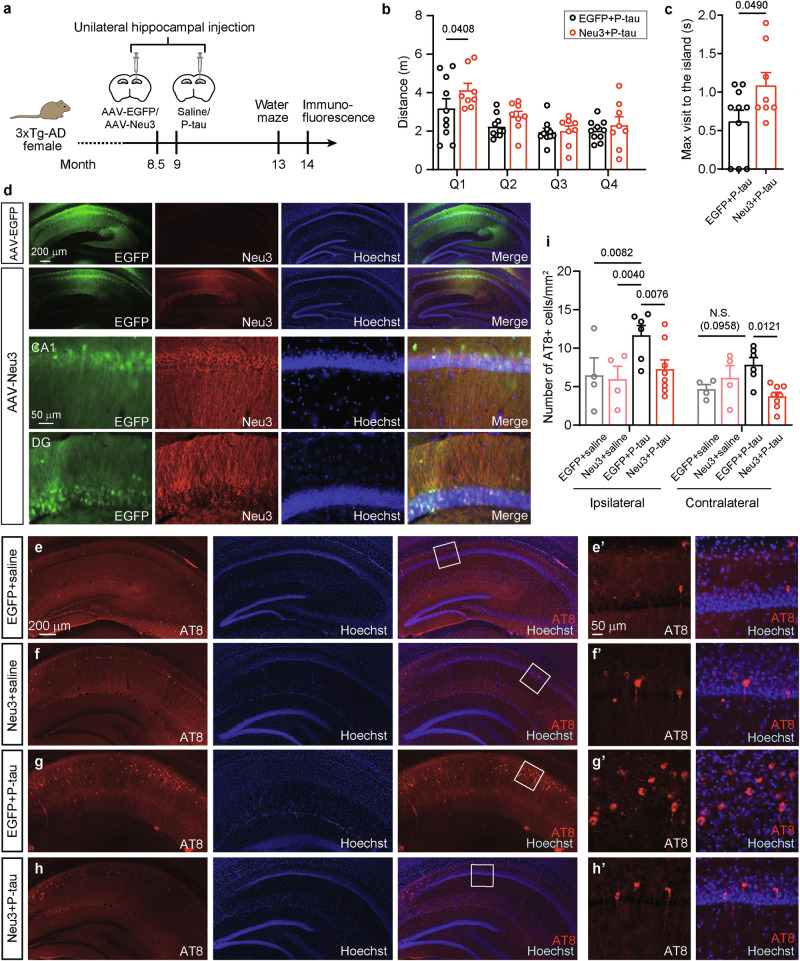
Neu3 improves spatial memory and mitigates tau pathology in AD P-tau-injected 3xTg-AD mice. **a** Experimental design flowchart. **b-c** The Morris water maze test was employed to assess spatial memory in AAV-EGFP + P-tau (EGFP + P-tau for short) and AAV-Neu3+P-tau (Neu3+P-tau for short) mice. **b** During the probe trial, the distance traveled by the mice in each quadrant was recorded (EGFP + P-tau, n = 10; Neu3+P-tau, n = 8) and analyzed by two-way ANOVA. Quadrant: *F*_3,64_ = 11.10, *P* < 0.0001; Neu3: *F*_1,64_ = 4.05_1_, *P* = 0.0483. **c** The maximum time spent visiting the island zone was recoded and analyzed using Student’s *t* test. **d** Immunofluorescence analysis of AAV-EGFP and AAV-Neu3-injected brains demonstrated overexpression of Neu3 in the hippocampus post-AAV administration. EGFP labels the virus expression. Hoechst stains the nuclei. **e-h** Mice brains underwent immunofluorescence using AT8 antibody (pS202/T205 tau) to visualize tau pathology. **e’-h’** Zoom**e**d**-**in views of the white box regions in **e-h**. **i** The dens**i**ty of AT8+ cells in mice hippocampus was quantified (EGFP+saline, n = 4; Neu3+saline, n = 4；EGFP + P-tau, n = 6；Neu3+P-tau, n = 8) and analyzed with two-way ANOVA. Side: *F*_1,36_ = 6.202, *P* = 0.0175; group: *F*_3,36_ = 5.976, *P* = 0.0021. N.S., not significant.

Next, to examine the effect of Neu3 on the transmission of AD P-tau-induced tau pathology, 8.5-month-old female 3xTg-AD mice received injections of either AAV-Neu3 or AAV-EGFP into the unilateral hippocampus. Two weeks later, AD P-tau was injected at the same site. By 13 months, the mice’s behavior was assessed using the water maze (Fig. 2a). During training, both the AAV-EGFP + P-tau and AAV-Neu3+P-tau groups took comparable amounts of time to find the escape platform (Supplementary Figure 3a). In the probe trial, no significant differences were observed in swimming speed between the two groups in the target quadrant (Supplementary Figure 3b). However, mice in the AAV-Neu3+P-tau group traveled significantly longer distances in the target quadrant (Fig. 2b) and spent more time in the target island zone (Fig. 2c), suggesting an improvement in spatial memory ability.

**Fig. 3:**
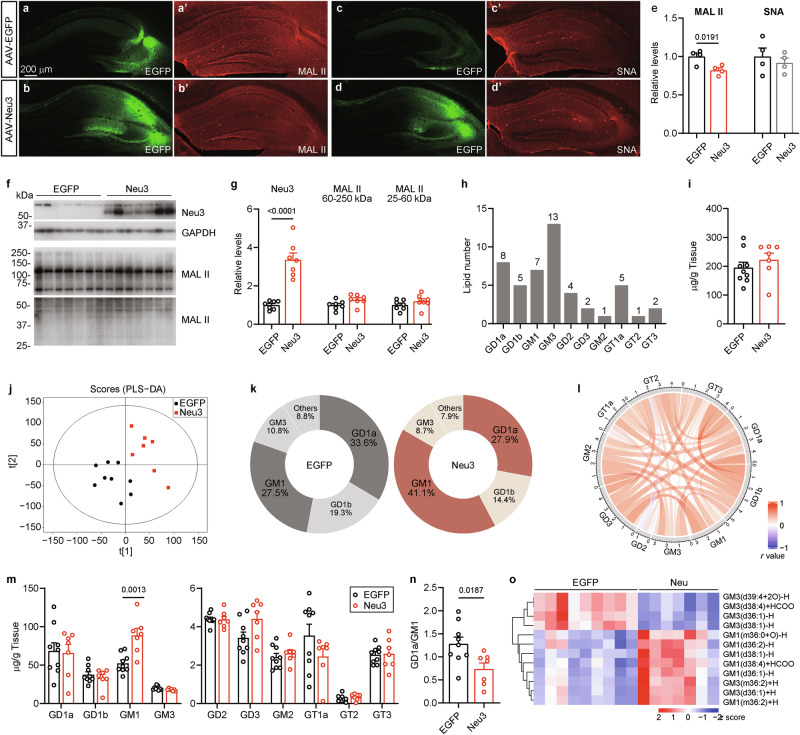
Neu3 enhances GM1 levels in mouse brain. **a-e** At 8.5 months of age, 3xTg-AD female mice received injections of AAV-EGFP (EGFP for short) or AAV-Neu3 (Neu3 for short) into the hippocampus. At 14 months, the brains were subjected to lectin staining. EGFP labels virus expression, while MAL II identifies α2-3 sialylation and SNA identifies α2-6 sialylation. **e** The relative intensity of MAL II or SNA in **a-d** was measured and analyzed by Student’s *t* test (n = 4). SNA, *P* = 0.5451. **f**, **g** The hippocampus was analyzed through Western blotting with antibodies against Neu3 and lectin blots using MAL II. GAPDH was used as loading control. The levels of Neu3 and MAL II were quantified and analyzed using Student’s *t* test (n = 7). MAL II (60-250 kDa), *P* = 0.0636; MAL II (25-60 kDa), *P* = 0.2514. **h-n** The gangliosides in the hippocampus of AAV-injected mice were analyzed via LC-MS/MS (EGFP, n = 9; Neu3, n = 7). **h** Number of gangliosides in each class. **i** Total ganglioside content in the hippocampus. Mann-Whitney test, *P* = 0.4079. **j** Partial Least Squares Discrimination Analysis (PLS-DA) of LC-MS/MS data was performed. **k** Proportion of each ganglioside class relative to total gangliosides. **l** Correlation analysis among ganglioside classes. **m** Quantitative analysis of ganglioside classes in the hippocampus. Student’s *t* test, GM1, *P* = 0.0013; GM3, *P* = 0.0573; GD2, *P* = 0.9172; GD3, *P* = 0.0548; GM2, *P* = 0.5145; GT2, *P* = 0.3786; GT3, *P* = 0.8738. Mann-Whitney test, GD1a, *P* = 0.7764; GD1b, *P* = 0.9182; GT1a, *P* = 0.3510. **n** The GD1a/GM1 ratio in mice hippocampus was calculated and analyzed by Student’s *t* test. **o** The heatmap of significantly altered gangliosides.

Brains from these mice were collected at 14 months of age and analyzed using immunofluorescence. High levels of Neu3 expression were detected in the hippocampus of mice injected with AAV-Neu3 (Fig. 2d), which was further confirmed by Western blotting for Neu3 (Fig. 3f, g). The AT8 antibody, which specifically targets tau phosphorylated at pS202/T205, was used to investigate tau pathology in the hippocampus (Fig. 2e-i). The AD P-tau injection significantly increased the density of AT8-positive (AT8 + ) cells in the ipsilateral hippocampus of mice treated with AAV-EGFP + P-tau (Figs. 2e, 2g, 2i); however, AAV-Neu3+P-tau injection notably reduced the density of AT8+ cells in that region (Figs. 2g, 2h, 2i), suggesting that Neu3 plays a role in inhibiting tau pathology triggered by AD P-tau injection. In the contralateral hippocampus, AAV-EGFP + P-tau mice exhibited a slight increase in AT8+ cell density compared to the AAV-EGFP+saline group, though this did not reach statistical significance (*P* = 0.0958, Fig. 2i). Notably, AT8 density was also significantly lower in the contralateral hippocampus of AAV9-Neu3+P-tau mice compared to AAV-EGFP + P-tau mice (Fig. 2i), indicating that Neu3 also helps to inhibit the spread of tau pathology from the injured side to the unaffected side. Additionally, immunofluorescence staining of these samples with T22, an antibody that targets tau oligomers, revealed a significant increase in T22 intensity in the mouse hippocampal region following AD P-tau injection (Supplementary Figure 3d-e). Consistent with the findings from AT8 staining, AAV-Neu3 significantly reduced the production of oligomeric tau induced by AD P-tau in both the ipsilateral and contralateral hippocampus (Supplementary Figure 3d-e). Overall, these findings suggested that overexpression of Neu3 in neurons can mitigate both the onset and the spread of tau pathology induced by AD P-tau.

### Neu3 enhances GM1 levels while reducing the GD1a/GM1 ratio in the hippocampus of 3xTg-AD mice

To examine the preferred sialyl linkage of Neu3 in the brain, lectin staining was conducted on the hippocampus of AAV-injected mice (Fig. 3a-e). This analysis revealed a decrease in α2-3 sialylation labeled by Maackia Amurensis lectin II (MAL II) (Figs. 3a, b, 3e), while α2-6 sialylation labeled by Sambucus Nigra lectin (SNA) remained unchanged (Fig. 3c-e). To determine whether the reduced MAL II staining was due to decreased α2-3 sialylation of glycoproteins or glycolipids, we conducted further investigations using MAL II for lectin blotting. The results showed minimal changes in α2-3 sialylation of hippocampal proteins, suggesting that the primary substrate of Neu3 may not be glycoproteins, which aligns with previous findings [46]. Gangliosides serve as substrates for Neu3 [46]. To explore the changes in gangliosides due to Neu3 overexpression in the brain, a liquid chromatography coupled to tandem mass spectrometry (LC-MS/MS) was performed (Fig. 3h-o). The results indicated no significant difference in the overall ganglioside content between the two mouse groups (Fig. 3i). Ten major classes of gangliosides were identified: GD1a, GD1b, GM1, GM3, GD2, GD3, GM2, GT1a, GT2, and GT3, comprising a total of 48 different ganglioside types (Fig. 3h-l). Notably, GD1a, GD1b, and GM1 accounted for over 80% of the total ganglioside content (Figs. 3k, 3m), aligning with the primary gangliosides present in the human brain [47].

As the most abundant gangliosides in the brain, GD1a represented 33.6%, while GM1 accounted for 27.5% of the total gangliosides detected in the hippocampus of AAV-EGFP mice (Fig. 3k), resulting in a GD1a/GM1 ratio of approximately 1.2. In contrast, the hippocampus of AAV-Neu3 mice exhibited a shift in this ratio to 0.7, with GM1 increasing to 41.1% and becoming the predominant ganglioside, while GD1a decreased to 27.9% (Fig. 3k). This change indicated a significant reduction in the GD1a/GM1 ratio (Fig. 3n). LC-MS/MS analysis revealed that 6 types of GM1 and 6 types of GM3 levels were significantly altered in AAV-Neu3 mice (Fig. 3o), yet only the total GM1 content in the hippocampus was significantly elevated (Fig. 3m), with other ganglioside classes showing no notable changes (Fig. 3m). Thus, overexpression of neuronal Neu3 results in elevated levels of GM1 and a decreased GD1a/GM1 ratio in the brain.

### Gangliosides GM1 and GD1a regulate AD P-tau-seeded tau aggregation

**Fig. 4:**
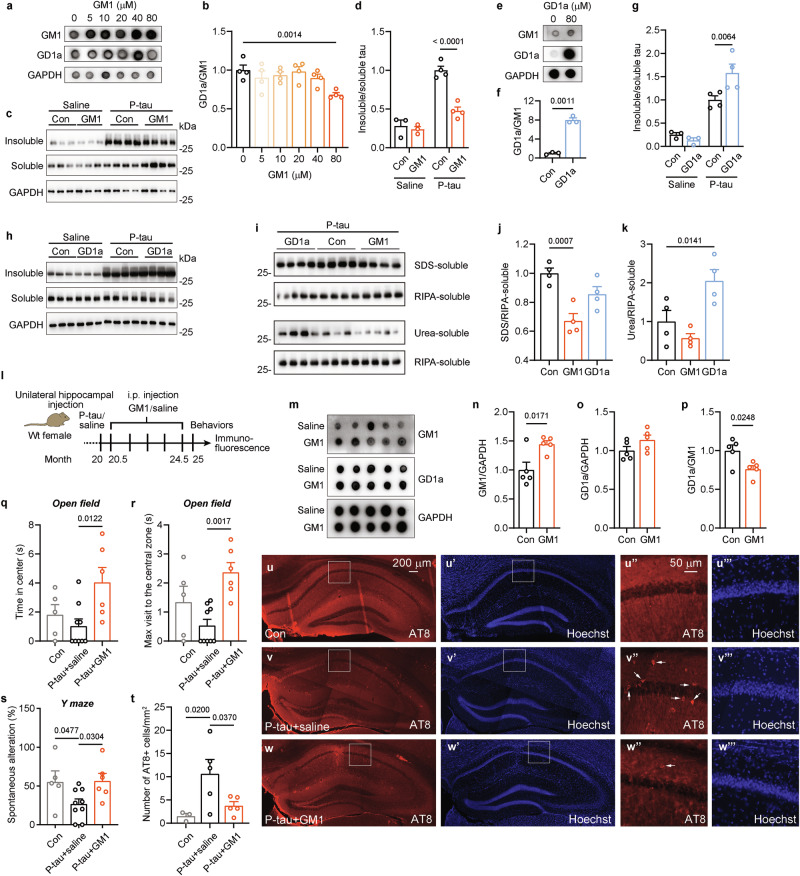
GM1 inhibits AD P-tau-seeded tau aggregation in cultured cells and in vivo and improves recognition in AD P-tau-injected mice. **a, b** HEK293T cells were treated with GM1 and lysed for dot blots. GAPDH was used as a loading control. **b** Quantification of GD1a/GM1 intensity in **a** was analyzed using one-way ANOVA (n = 4), *F* = 3.688, *P* = 0.0179. **c, d** HEK293T cells were transfected with pCI/HA-tau_151-391_, treated with 80 μM GM1 for 24 h, and induced with AD P-tau for tau aggregation. The RIPA-insoluble and soluble fractions were assessed via Western blot using anti-HA antibodies. GAPDH in RIPA-soluble fractions served as a loading control. **d** The ratio of insoluble to soluble tau in **c** was quantified (Con+saline, n = 3; GM1+saline, n = 3; Con+P-tau, n = 4; GM1 + P-tau, n = 4) and analyzed by two-way ANOVA. P-tau: *F*_1,10_ = 74.97, *P* < 0.0001; GM1: *F*_1,10_ = 26.22, *P* = 0.0005; *P*-tau × GM1: *F*_1,10_ = 18.9_1_, *P* = 0.0014. **e**, **f** HEK293T cells were treated with 80 μM GD1a for 24 h. Dot blot was performed to detect GD1a levels. f Quantification of GD1a/GM1 ratio in e was analyzed using Student’s *t* test (n = 3). **g**, **h** HEK293T cells were transfected with pCI/HA-tau_151-391_, treated with 80 μM GD1a for 24 h, followed by AD P-tau induction. The RIPA-insoluble and soluble fractions were subjected to Western blot using anti-HA antibodies. Quantification of the insoluble to soluble tau ratio was analyzed by two-way ANOVA. P-tau: *F*_1,10_ = 73.24, *P* < 0.0001; GD1a: *F*_1,10_ = 3.163, *P* = 0.1057_;_ P-tau × GD1a: *F*_1,10_ = 7.385, *P* = 0.02_1_7_._ (Con+saline, n = 3; GD1a+saline, n = 3; Con+P-tau, n = 4; GD1a+P-tau, n = 4). **i**, **j** HEK293T cells were transfected with pCI/HA-tau_151-391_, treated with 80 μM GM1 or GD1a for 24 h, followed by AD P-tau induction for tau aggregation. The resulting SDS-soluble, urea-soluble, and RIPA-soluble fractions were analyzed via Western blotting using anti-HA antibodies. **j** Quantification of the ratio of SDS-soluble to RIPA-soluble tau (n = 4) was analyzed by one-way ANOVA, *F* = 12.55, *P* = 0.0025. **k** Quantification of the ratio of urea-soluble to RIPA-soluble tau (n = 4) was analyzed by one-way ANOVA, *F* = 9.653, *P* = 0.0058. **l** Experimental design flowchart. **m** Brain samples were collected 2 days post-GM1 injection and subjected to dot blot (n = 5). **n** Relative levels of GM1 were quantified and analyzed using Student’s *t* test. **o** Relative levels of GD1a were quantified and analyzed using Student’s *t* test (*P* = 0.1284). **p** The ratio of GD1a to GM1 was calculated and analyzed using Student’s *t* test. **q** The duration mice spent in the central zone of the open field was measured and analyzed using Kruskal-Wallis test (*P* = 0.0367) followed by uncorrected Dunn’s test. **r** Maximum visit time in the central zone of the open field was recorded and analyzed using Kruskal-Wallis test (*P* = 0.0025) followed by uncorrected Dunn’s test. Con (both hippocampus and intraperitoneal injections of saline), n = 5; P-tau+saline, n = 9; P-tau+GM1, n = 6. **s** The percentage of spontaneous alteration in the Y-maze was recorded and analyzed using one-way ANOVA, *F* = 3.687, *P* = 0.0468. Con, n = 5; P-tau+saline, n = 9; P-tau+GM1, n = 6. t-w Mouse brains underwent immunofluorescence with AT8 antibody to visualize tau pathology. **t** The density of AT8+ cells in the hippocampus in u-w was quantified (Con, n = 3; P-tau+saline, n = 5; P-tau+GM1, n = 5) and analyzed with one-way ANOVA. *F* = 4.719, *P* = 0.0360. AT8 labels pS202/T205 tau. Hoechst labels the nuclei. u”-w” Zoomed-in views of the regions marked by white boxes in u-w. u”’-w”’ Zoomed-in views of the regions marked by white boxes in u’-w’. Arrow, AT8+ cells.

To analyze the roles of Neu3 substrate GD1a and its product GM1 in tau aggregation, the effects on AD P-tau-seeded tau aggregation in HEK293T cells were investigated. The cells were initially treated with various concentrations of GM1. Dot blot analysis revealed that 80 μM GM1 significantly elevated GM1 levels while exerting minimal influence on GD1a content (Supplementary Figure 4a-b), resulting in a reduced GD1a/GM1 ratio (Fig. 4a, b). Subsequently, the cells were transfected with pCI/HA-tau_151-391_ and exposed to AD P-tau to trigger aggregation. Notably, the co-administration of 80 μM GM1 with AD P-tau led to a significant reduction in the ratio of insoluble tau fractions (Supplementary Figure 4c-d). In contrast, treatment with 80 μM GD1a increased GD1a levels while preserving GM1 levels (Supplementary Figure 4e-f), thereby raising the GD1a/GM1 ratio (Fig. 4e, f). Surprisingly, co-treatment of AD P-tau with GD1a did not yield significant changes in the insoluble/soluble tau ratio (Supplementary Figure 4g-h).

**Fig. 5:**
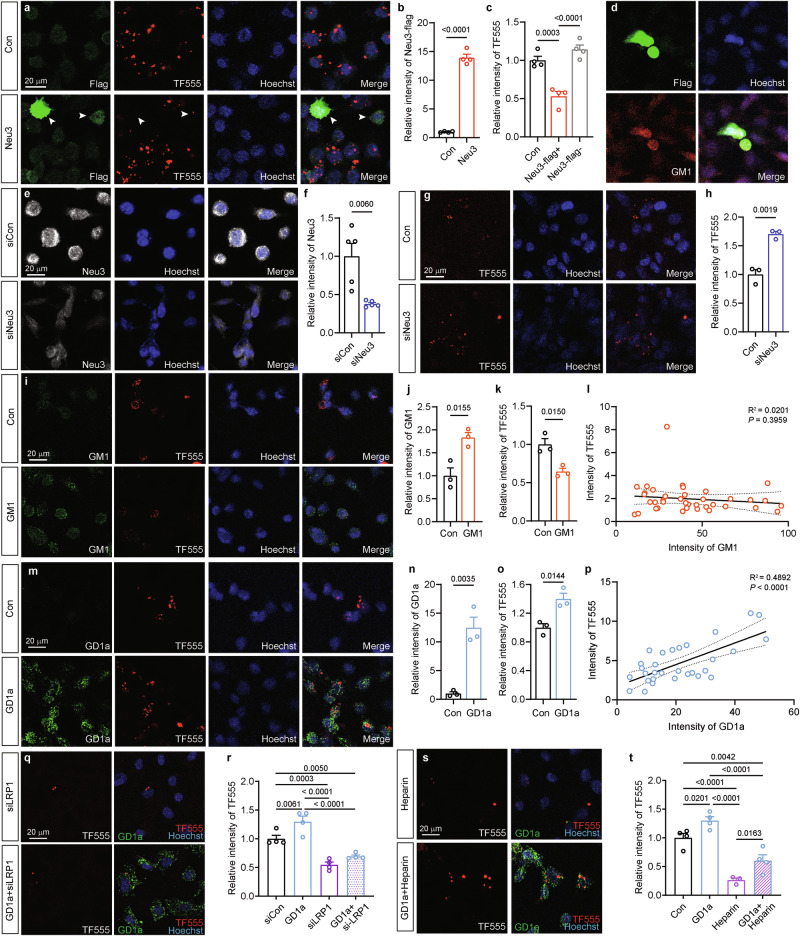
Neu3 and the gangliosides GD1a and GM1 regulate the internalization of tau aggregates. **a** SH-SY5Y cells were transfected with pcDNA3.1/Neu3 containing a flag tag, followed by treatment with TF555 and analyzed via immunofluorescence. Hoechst stains the nuclei. Arrow head, Neu3-positive cells. **b** Quantification of Neu3-flag fluorescence intensity in **a** was analyzed using Student’s *t* test (n = 4). **c** Quantification of TF555 intensity in control group, Neu3-flag positive and negative cells from Neu3-transfected groups in **a** was analyzed using one-way ANOVA (n = 4), *F* = 31.42, *P* < 0.0001. **d** SH-SY5Y cells were transfected with pcDNA3.1/Neu3 and subjected to immunofluorescence using anti-flag and anti-GM1 antibodies. **e** Following transfection with siNeu3, SH-SY5Y cells were immunostained with anti-Neu3 antibody. **f** Quantification of Neu3 intensity in **e** was analyz**e**d using Student’s *t* test (n = 5). g SH-SY5Y cells transfected with siNeu3 were treated with TF555. **h** Quantification of TF555 intensity in **g** was analyzed using Student’s *t* test (n = 3). **i** SH-SY5Y cells were treated with 80 uM GM1, followed by exposure to TF555, and subsequent immunostaining with anti-GM1 antibodies. **j** Quantification of GM1 intensity in **i** was analyzed using Student’s *t* test (n = 3). **k** The intensity of TF555 in **i** was quant**i**fied with Student’s *t* test (n = 3). **l** Pearson correlation of GM1 intensity and TF555 intensity (n = 38). m SH-SY5Y cells treated with 80 uM GD1a were incubated with TF555 and immunostained with anti-GD1a antibodies. **n** Quantification of GD1a intensity in **m** was analyzed using Student’s *t* test (n = 3). **o** Quantification of TF555 intensity in **m** was analyzed using Student’s *t* test (n = 3). **p** Pearson correlation analysis between GD1a intensity and TF555 intensity (n = 30). Arrow, uptake of TF555. **q-r** SH-SY5Y cells transfected with siCon or siLRP1 were incubated with or without GD1a, followed by exposure to TF555 (figures for siCon and GD1a were not shown). **r** Quantification of TF555 intensity was analyzed by two-way ANOVA. GD1a: *F*_1,12_ = 12.29, *P* = 0.0043; siLRP1: *F*_1,12_ = 69.74, *P* < 0.0001; GD1a × siLRP1: *F*_1,12_ = 1.430, *P* = 0.2548 (n = 4). **s-t** SH-SY5Y cells were incubated with or without either GD1a or heparin, followed by exposure to TF555 (figures for Con and GD1a were not shown). **t** Quantification of TF555 intensity was analyzed by two-way ANOVA. GD1a: *F*_1,11_ = 15.40, *P* = 0.0024; heparin: *F*_1,11_ = 77.53, *P* < 0.000_1;_ GD1a × heparin: *F*_1,11_ = 0.0554, *P* = 0.8183 (n = 4).

**Fig. 6:**
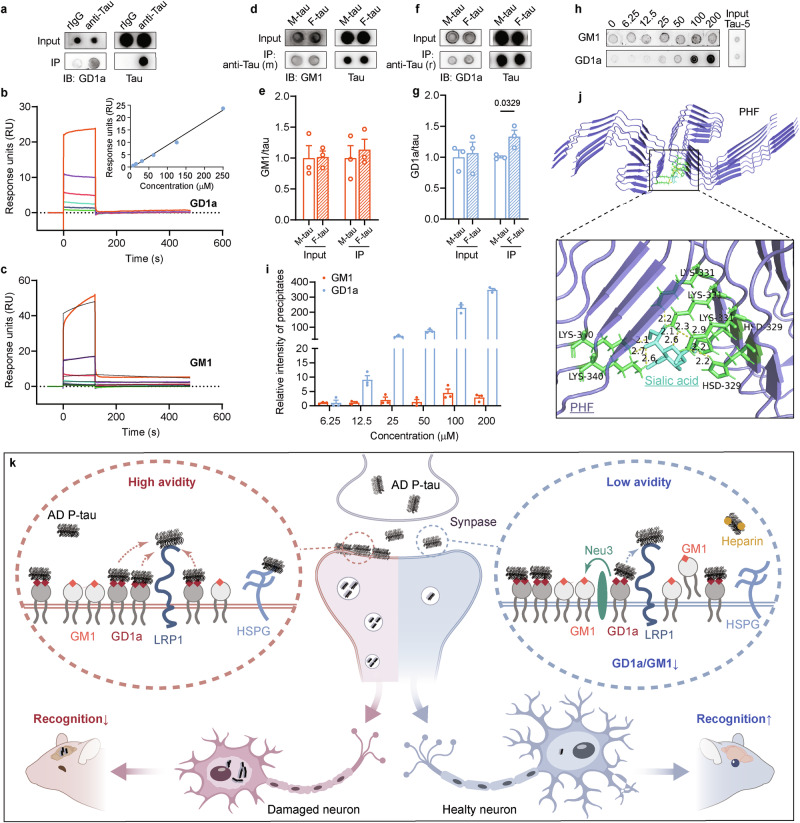
GD1a exhibits superior avidity for tau aggregates compared to GM1. **a** GD1a and recombinant tau_151-391_ protein were incubated and subjected to pull-down assay utilizing rabbit anti-Tau antibodies,with rabbit IgG (rIgG) as a control. The input samples and precipitates are analyzed by dot blot using mouse anti-GD1a and anti-Tau (HT7) antibodies. **b** SPR assay characterizing the interaction between GD1a and tau_151-391_. GD1a concentrations used were 7.8 μM, 15.6 μM, 31.2 μM, 62.5 μM, 125 μM, and 250 μM (arranged from bottom to top). Inset, SPR data were fitted to a 1:1 Langmuir binding model. **c** SPR analysis characterizing the specific binding of GM1 to tau_151-391_, with GM1 concentrations of 15.6 μM, 31.2 μM, 62.5 μM, 125 μM, 250 μM, and 500 μM (arranged from bottom to top). Colour lines, SPR data from varying analyte concentrations. Black lines, data were fitted to a steady-state model. **d** Equal amounts of monomeric tau_151-391_ (M-tau) and fibrillated tau_151-391_ (F-tau) were incubated with GM1 and then subjected to a pull-down assay using mouse (m) anti-tau antibody HT7. **e** The levels of input and pulled-down GM1 (normalized to tau) from **d** were calculated and analyzed using Student’s *t* test (n = 3). Input, *P* = 0.9373; IP, *P* = 0.6277. **f** Equal amounts of M-tau and F-tau were incubated with GD1a and then subjected to a pull-down assay using rabbit (r) anti-tau antibodies. **g** The levels of input and pulled-down GD1a (normalized to tau) from **f** was calculated and analyzed using Welch’s *t* test (n = 3). Input, *P* = 0.7718. **h** F-tau were incubated with GM1 or GD1a, followed by centrifugation. The resulting pellets were subjected to dot blots. **i** The levels of GM1 and GD1a from **h** was measured. **j** Docking interactions between AD PHF tau (purple) and sialic acid (blue) are depicted. Contacting residues are presented in stick representation (green). Yellow dashed lines, hydrogen bonds. **k** Left panels, GD1a on the cell membrane mediates the adhesion of AD P-tau via additional sialic acid, facilitates AD P-tau internalization through LRP1 and contributes to the spread of tau pathology. Right panels, Overexpression of Neu3, which removes one sialic acid from GD1a, or the administration of GM1 both decrease the GD1a/GM1 ratio at the cell membrane. This change reduces the local concentration of AD P-tau recruited by GD1a, thereby decreasing the uptake of AD P-tau by LRP1. This inhibition of tau pathology transmission results in a protective effect against cognitive impairment related to AD P-tau.

To role out the potential interaction between the gangliosides and AD P-tau, which may interfere with the internalization of AD P-tau and subsequently affect AD P-tau-induced tau aggregation, we pre-treated the cells with GM1 or GD1a for 24 h before introducing AD P-tau to induce tau aggregation for 42 h. This approach revealed that GM1 pre-treatment still reduced the ratio of insoluble tau, similar to its effect when co-administered with AD P-tau (Fig. 4c, d), suggesting that membrane-bound GM1, rather than free GM1, mediates AD P-tau-induced tau aggregation. However, unlike co-treatment with AD P-tau, pre-treatment with GD1a significantly increased the ratio of insoluble tau (Fig. 4g, h). We speculate that when free GD1a is added simultaneously with AD P-tau, it may interact with AD P-tau (Fig. 6h-j) and competitively inhibit its binding to GD1a on the cell membrane. This interaction could hinder the uptake of AD P-tau into the cells, limiting tau aggregation. In contrast, GD1a pre-treatment effectively altered GD1a content on the cell membrane, facilitating the entry of AD P-tau (Fig. 5m-p) and resulting in increased tau aggregation.

To explore the effects of GM1 and GD1a on the levels of SDS-soluble and urea-soluble tau induced by AD P-tau, we overexpressed pCI/HA-tau_151-391_ in HEK293T cells and treated them with 80 μM GM1 or GD1a for 24 h, followed by the induction of tau aggregation using AD P-tau. Forty-two hours after the addition of AD P-tau, the cells were lysed with RIPA buffer and subjected to ultracentrifugation. The pellet was collected and subsequently dissolved in either SDS buffer or urea buffer. Western blot analysis of tau levels in each fraction revealed that GM1 significantly reduced the amount of SDS-soluble tau but had no effect on urea-soluble tau (Fig. 4i-k), similar to the roles of Neu3. Surprisingly, GD1a treatment had a limited impact on SDS-soluble tau while markedly increasing the level of urea-soluble tau (Fig. 4i-k). We speculate that this may be attributed to exogenous GD1a inducing a more substantial increase in the GD1a/GM1 ratio (Fig. 4e, f) in comparison to siNeu3 treatment (Figs. 1l, 1n), ultimately facilitating more pronounced tau filament formation. Taken together, these findings suggest that GM1 inhibits AD P-tau-induced tau aggregation, whereas GD1a promotes it.

### GM1 inhibits tau pathology and improves recognition in AD P-tau-injected mice

To investigate whether GM1 inhibits tau pathology seeded by proteopathic tau in vivo, AD P-tau was unilaterally injected into the hippocampus of 20-month-old wild-type mice, minimizing the impact of GM1 on the cleavage of transgenic APP [23]. After 2 weeks, the mice received intraperitoneal injections of GM1 at a dosage of 50 mg/kg for 5 consecutive days, once a month for 5 months (Fig. 4l). The dot blot analysis revealed an increase in GM1 levels and a decrease in the GD1a/GM1 ratio in the hippocampus of the mice that received GM1 injections (Fig. 4m-p). The open field test demonstrated that P-tau+GM1 mice spent significantly more time, as well as had longer maximum visit durations, in the center zone compared to P-tau+saline mice (Fig. 4q-r, Supplementary Figure 4i). Additionally, P-tau+GM1 mice exhibited a marked increase in the percentage of spontaneous alterations in the Y-maze (Fig. 4s, Supplementary Figure 4j), suggesting improvements in recognition and short-term spatial memory. No significant differences were observed in the water maze test (Supplementary Figure 4k), likely because the lower tau expression and less pronounced AD P-tau-induced pathology in wild-type mice (Figs. 4v, 2g), which may have obscured the potential protective effects of GM1 on water maze performance. Nevertheless, immunostaining revealed that GM1 treatment notably reduced the density of AT8+ cells in the hippocampus of mice injected with AD P-tau (Fig. 4u-w). These results suggest that GM1 has an inhibitory effect on tau aggregation, similar to Neu3, and alleviates recognition defects in AD P-tau injected mice.

### Neu3-regulated gangliosides modulate the internalization of tau aggregates

Neu3 is a membrane-bound sialidase [32] that converts GD1a to GM1, both of which are key components of the cell membrane. It has been reported that highly sialylated gangliosides like GD1a are associated with increased transferrin internalization, whereas Neu3 impairs the endocytosis of transferrin [32]. This raises the question of whether the gangliosides regulated by Neu3 might play a role in the uptake of tau aggregates, potentially influencing the development and spread of tau pathology. To explore this hypothesis, the roles of Neu3, as well as the gangliosides GM1 and GD1a, were examined in relation to the internalization of tau aggregates. Overexpression of Neu3 in human neuroblastoma SH-SY5Y cells significantly increased the cellular level of GM1 (Fig. 5d, Supplementary Figure 5c) and resulted in a decreased GD1a/GM1 ratio (Supplementary Figure 5a-d), while siNEU3 markedly decreased GM1 levels and raised the GD1a/GM1 ratio (Supplementary Figure 5e-h). In vitro fibrillization of recombinant tau_151-391_ was induced, and the resulting filaments were labeled with Alexa Fluor 555 Dye (TF555) prior to being added to the cells. Overexpression of Neu3 significantly inhibited the uptake of TF555 (Fig. 5a-c), whereas siNEU3 markedly enhanced TF555 internalization (Fig. 5g, h). Additionally, treatment with 80 μM GM1 was observed to reduce the uptake of TF555 (Fig. 5i-k), while 80 μM GD1a enhanced its internalization (Fig. 5m-o). Notably, the intensity of GD1a (Fig. 5p), but not GM1 (Fig. 5l), showed a positive correlation with the intensity of internalized TF555. These findings suggest that Neu3-mediated gangliosides, with GD1a as a key molecule, play a crucial role in modulating the internalization of tau aggregates.

LRP1 and HSPG are recognized tau receptors [48]. To investigate the relationship between GD1a and these known tau receptors, we transfected SH-SY5Y cells with siLRP1 or treated them with anti-LRP1 antibodies, alongside GD1a treatment. Both the siLRP1 (Fig. 5q, r, Supplementary Figure 5i, j) and antibody blockade (Supplementary Figure 5k-l) significantly diminished TF555 internalization. Importantly, GD1a treatment did not reverse these impaired processes (Fig. 5q, r, Supplementary Figure 5k-l), indicating that the GD1a-mediated internalization of tau aggregates is dependent on LRP1.

Next, we treated the cells with 20 µg/mL heparin to inhibit HSPG-mediated tau endocytosis [12], alongside GD1a treatment. The results demonstrated that heparin significantly suppressed TF555 internalization, while GD1a treatment notably restored the levels of internalized TF555 (Fig. 5s, t), suggesting that GD1a may act as a functional alternative pathway for HSPG-mediated internalization of tau.

### GD1a exhibits a stronger avidity for tau filaments compared to GM1

GD1a and GM1 are well-established receptors for bacteria and viruses [26, 28, 29]. This raised the question of whether these gangliosides could also bind to tau and mediate its uptake. To investigate this, pull-down assay was conducted to determine if GD1a and GM1 interact with tau. A concentration of 10 µg/mL of tau_151-391_ monomers were incubated with 6.4 µM of either GD1a or GM1 for 2 h, employing tau antibodies for the pull-down assay. Immunoblotting results demonstrated that GD1a could be pulled-down by tau_151-391_ monomers (Fig. 6a). However, GM1 displayed non-specific binding with IgG (data not shown), which is why this similar assay was not carried out. To further investigate the interactions, surface plasmon resonance (SPR) analysis was employed. The results indicated that GM1 exhibited moderate specific binding to tau_151-391_, with a dissociation constant (*k*_*D*_) of 6.453×10^-5 ^M (Fig. 6c). In contrast, GD1a exhibited a linear increase in binding to tau_151-391_ across the concentration range of 7.8 μM to 250 μM (Fig. 6b). This suggests that the interaction between GD1a and tau monomer does not follow a typical specific binding pattern, indicating low affinity and non-specific binding.

Similarly, it has been reported that the dimeric S1 protein of the Middle East respiratory syndrome coronavirus (MERS-CoV) has low affinity for sialoglycans. In contrast, the multivalent S1 protein engages with sialoglycans through a binding pattern characterized by high specificity, low affinity but high avidity [49]. Inspired by this, we investigated whether the tau protein similarly requires multivalent presentation (i.e., in an aggregated form) to achieve high-avidity binding with sialic acids on gangliosides. Equal amount of tau_151-391_ monomer (M-tau) and heparin-induced filament (F-tau) were incubated with GD1a or GM1 for 2 h and then subjected to pull-down assay using tau antibodies (Fig. 6d-g). The results from immunoblotting showed that both M-tau and F-tau precipitated comparable amounts of GM1 (Fig. 6d, e). However, F-tau precipitated significantly more GD1a than M-tau (Fig. 6f, g), suggesting that GD1a, but not GM1, exhibits a preference for binding to aggregated tau.

Potential differences in the binding of GD1a and GM1 to tau aggregates were further examined (Fig. 6h, i). Tau_151-391_ filament was incubated with 6.25 μM to 200 μM of GD1a or GM1 for 2 h. After ultracentrifugation at 130000 × g, the aggregates were collected and subjected to dot blot analysis to quantify GD1a and GM1 content in the pellet. It was observed that with increasing concentration, tau filament bound to GD1a increased dramatically by over 348 times. In contrast, GM1 displayed minimal change, with a maximum binding increase of only 4.44 times at 100 μM. These findings highlight that while both gangliosides GD1a and GM1 can bind to tau, GD1a demonstrates a significantly stronger avidity for tau aggregates in comparison to GM1. These findings imply that the interaction between GD1a and tau does not conform to the typical specific binding analyzed by SPR but is instead more likely influenced by a multivalency-driven, high-avidity binding.

## Discussion

This study highlights the significant role of Neu3 and GM1 in decreasing the GD1a/GM1 ratio, which helps inhibit AD P-tau-induced tau pathology aggregation and transmission. The findings demonstrate this effect both in vitro and in vivo, while also showing an improvement in cognitive deficits in AD mice. The research reveals that both GD1a and GM1 interact with tau protein; however, GD1a shows a greater avidity for tau aggregates compared to GM1. Moreover, higher levels of GD1a on cell membranes are correlated to increased internalization of tau aggregates, with GD1a-mediated uptake of tau occurring via an LRP1-dependent mechanism. We speculate that GD1a, characterized by its two sialic acid groups, specifically facilitates the concentration of AD P-tau to the plasma membrane, subsequently promoting its internalization through the LRP1 pathway (Fig. 6k). Interestingly, neither the expression of Neu3 nor the administration of GM1 altered the total levels of GD1a in vivo (Figs. 3m, 4o). This suggests that a reduction in the proportion of GD1a among membrane gangliosides is sufficient to diminish tau internalization. We hypothesize that this happens because the lower GD1a/GM1 ratio results in a reduced local concentration of GD1a within the functional vicinity of LRP1 (Fig. 6k). Consequently, fewer tau molecules are recruited to each individual receptor, which ultimately leads to a decrease in LRP1-mediated tau endocytosis. On the other hand, GD1a molecules that are situated outside the functional vicinity of the receptors can still recruit tau, but they cannot independently trigger the internalization process. This combined effect contributes to the overall reduction in tau internalization, which may potentially impede the propagation of tau pathology (Fig. 6k).

The role of GM1 and Neu3 in AD remains controversial [47]. Some studies indicate that GM1 may exacerbate AD by binding to Aβ, promoting the formation of Aβ fibrils, and facilitating their interaction with cell membranes [50–53]. Expression of AAV2/8-CMV-Neu3 in the hippocampus [23] or peripheral GM1 delivery [54] in APP/PS1 mice leads to increased Aβ plaque accumulation and impaired spatial learning. Our study using AAV9-hSyn-Neu3 showed no significant changes in Aβ plaque intensity in the hippocampus (Supplementary Figure 3c). We postulate that the neuron-specific expression of Neu3 via synapsin 1 promoter may have restricted its impact on Aβ pathology in 3xTg-AD mice. Conversely, some findings indicate that GM1 and Neu3 could offer protective effects against AD [55]. For example, the surface-exposed sugar groups of GM1 interact with Aβ_42_, reducing its propensity to adopt a β-sheet conformation [22]. In rat hippocampal neurons, Neu3-mediated GM1 upregulation enhances neuronal polarity and axonal formation through increased TrkA receptor activity [56]. Although early clinical trials of GM1 via intramuscular injection for 6 weeks were inconclusive in cognitive improvement [20], a 2002 study involving continuous GM1 injection into the anterior horn of the lateral ventricle in 5 early-onset AD patients led to notable enhancements in motor performance and neuropsychological assessments [21]. Based on our results, it’s likely that the variability in GM1’s effects in AD patients [18] may stem from its differential regulation of tau and Aβ pathologies.

Our results also showed that Neu3’s effects on the behavior of AD model mice not injected with AD P-tau were minimal (Supplementary Figure 2), indicating that Neu3 overexpression primarily affects the uptake and propagation of AD P-tau, rather than reversing the impairments caused by the overexpression of tau mutants. In human AD, genetically inherited cases comprise only about 1% of all instances, with the vast majority being sporadic AD. Unlike the toxic pathways observed in transgenic animal models due to short-term and high-level tau overexpression, the tau pathology in sporadic AD is marked by the interneuronal transfer of pathological tau, leading to functional impairments in recipient neurons [57, 58]. Thus, the mechanism of Neu3—intervening in the uptake and spread of pathological tau—targets this critical aspect, suggesting that Neu3 might be of greater relevance to sporadic AD.

Gangliosides play a crucial role in the endocytosis of a variety of proteins [32, 59], viruses [28], and bacteria [29]. GD1a has been shown to associate with and function as a co-receptor for several molecules, including neurotoxins [60] and Toll-like receptors [59]. Our findings indicate that the GD1a-mediated internalization of tau aggregates is reliant on the well-characterized tau receptor LRP1 [13] (Fig. 5q, r, Supplementary Figure 5i, j), suggesting that GD1a likely acts as a co-receptor for LRP1. In this proposed model (Fig. 6k), GD1a may enhance the presence of tau aggregates on the plasma membrane through its interaction with them, thereby facilitating their subsequent uptake via LRP1.

Another tau receptor, HSPG, is known to mediate tau internalization through two primary pathways: HSPG-triggered macropinocytosis and an HSPG-facilitated pathway, in which HSPG-bound tau associates with LRP1 for clathrin-mediated endocytosis [48]. Notably, GD1a treatment restored the levels of internalized TF555 in the cells treated with heparin (Fig. 5s, t), indicating that GD1a serves a complementary function to HSPG. Given that GD1a-mediated tau uptake is dependent on LRP1, we infer that GD1a cannot independently mediate tau internalization in the same manner as HSPG-triggered macropinocytosis. A more plausible scenario is that GD1a may compete with heparin for binding to tau aggregates, or it could bind to tau aggregates that are already associated with heparin, compensating for the inhibited HSPG function and facilitating their internalization via the LRP1-mediated pathway. In this context, GD1a provides an alternative route when the canonical HSPG-dependent entry is obstructed.

However, it remains unclear whether GD1a and heparin compete for binding to tau. HSPG possesses highly negatively charged heparan sulfate glycosaminoglycan chains [48, 61, 62]. The binding of tau aggregates to neuronal surface HSPG relies on specific heparin-binding motifs within tau, enriched with positively charged amino acids such as Lys, Arg, and His [48]. Sialic acids, which are negatively charged nine-carbon monosaccharides [63], play a crucial role in GD1a’s binding to tau. Theoretically, GD1a could interact with tau through similar charge-based mechanisms involving its sialic acids. Due to technical limitations, this study was unable to experimentally confirm the binding sites and modes of GD1a to tau. Also, the flexibility of ganglioside conformers made it challenging to obtain a suitable three-dimensional structure for molecular docking. Nevertheless, the presence of an extra sialic acid on GD1a’s terminal galactose enhances its avidity for tau aggregates compared to GM1. Additionally, previous X-ray crystallography has revealed that the two terminal sialic acids of GD1a dock into the receptor-recognizing knob domain of the Adenovirus type 37 fiber protein [26]. This led us to hypothesize that sialic acids may bind to the alkaline microtubule binding domain (MTBD) of tau [64], thereby mediating the interaction between gangliosides and tau. To explore this, we conducted molecular docking between ganglioside monosaccharides and the paired helical filament (PHF) from AD brain (Protein Data Bank ID: 8BGV, 3.27 Å) [65]. Notably, an interaction was formed between sialic acid and the protofilament interface of PHF, involving amino acids His329, Lys331, and Lys340 within the third and forth microtubule-binding repeats, with an affinity of -4.7 kcal/mol (Fig. 6j). The interactions of the remaining monosaccharides from GD1a and GM1 glycans—galactose, GalNAc, and glucose—with PHF occur outside of the protofilament interface and exhibit less favorable free energy (-4.3 kcal/mol, -4.5 kcal/mol, and -4.2 kcal/mol, respectively) (Supplementary Figure 6). These results indicate that the sialic acid component of ganglioside oligosaccharides may play a primary role in modulating ganglioside binding to tau. Future research is needed to definitively map the binding sites between GD1a (and its sialic acid moieties) and tau, which will provide clearer insights into whether GD1a and HSPG compete for tau binding.

In conclusion, this study reveals that ganglioside GD1a acts as a co-receptor facilitating the internalization of tau, while reducing ganglioside sialylation inhibits tau uptake, blocks the propagation of tau pathology, and enhances cognitive functions in AD model mice. These findings help clarify the complex roles of gangliosides in AD and suggest that further investigation into the neuroprotective effects of ganglioside oligosaccharides [22, 66] may provide a promising new strategy for AD treatment.

## Supplementary information

SUPPLEMENTAL MATERIAL

## Data Availability

Supplementary information is available on the MP’s website. Data can be requested by contacting the corresponding author (D.C.).
