# Supplementary material for: Ganglioside sialylation modulates tau internalization and pathology spread
Source: Mol Psychiatry. 2025 Dec 15;31(5):2561–74. doi: 10.1038/s41380-025-03394-2 (PMC13099399; doi:10.1038/s41380-025-03394-2)
Supplement: Supplementary file 1 — SUPPLEMENTAL MATERIAL [file 41380_2025_3394_MOESM1_ESM.doc]

**Supplementary Figures**

**Supplementary Figure 1 Regulation of Neu3 Expression Levels via transfection of pcDNA3.1/Neu3 or siNeu3.** **a** HEK293T cells were transfected with pcDNA3.1/Neu3, followed by Western blot analysis utilizing anti-Neu3 antibodies. GAPDH was included as a loading control. **b** The intensity of Neu3 was normalized to GAPDH levels in **a** (n = 3) and analyzed using **Student’s *t* test**. **c** HEK293T cells were transfected with siNeu3 and subjected to Western blots with anti-Neu3 antibodies. **d** The intensity of Neu3 in **c** was normalized to GAPDH levels (n = 3) and analyzed using **Student’s *t* test**. **e** HeLa cells were co-transfected with pCI/HA-tau_151-391_ and pCMV3/Neu2. Tau aggregation was induced with AD P-tau, and cells were analyzed through immunofluorescence using anti-HA antibodies. The quantification of HA-positive cells containing tau aggregates was performed (n = 4). **T**wo-way ANOVA, P-tau: *F*_1,12_ = 17.29, *P* = 0.0013; Neu2: *F*_1,12_ = 0.2770, *P* = 0.6083. **f** HEK293T cells were transfected with Neu3, and the cell lysates were analyzed via dot blot analysis. Quantification of GM1/GAPDH (n = 4) was analyzed by **Student’s *t* test,** *P* = 0.8450**. g** Quantification of GD1a/GAPDH in Neu3-expressed cells (n = 4) was analyzed by **Student’s *t* test. h** HEK293T cells were transfected with siNeu3, and the cell lysates were analyzed via dot blot analysis. Quantification of GM1/GAPDH (n = 3) was analyzed by **Student’s *t* test. i** Quantification of GD1a/GAPDH in siNeu3-expressed cells (n = 3) was analyzed by **Student’s *t* test,** *P* = 0.1181**. j** HEK293T cells were co-transfected with pCI/HA-tau_151-391_ and Neu3 or siNeu3, followed by AD P-tau induction for tau aggregation. The resulting urea-soluble and RIPA-soluble fractions were analyzed via Western blotting using anti-HA antibodies. **k** Quantification of the ratio of urea-soluble to RIPA-soluble tau in Neu3-expressed cells (n = 4) was analyzed by **Student’s *t* test,** *P* = 0.6672**. l** Quantification of the ratio of urea-soluble to RIPA-soluble tau in siNeu3-treated cells (n = 4) was analyzed by Mann-Whitney test**,** *P* = 0.8857**.**

**Supplementary Figure 2 Neu3 has no significant effects** **on the Morris water maze performance of 3xTg-AD and PS19 mice. a** Experimental design flowchart. At 6 months of age, 3xTg-AD female underwent unilateral injection of either AAV-EGFP or AAV-Neu3 into the lateral ventricle. **b-e** The Morris water maze test was conducted to evaluate cognitive function at 12 months of age. **b** During the training phase, the latency (time taken to reach the escape platform) was recorded and analyzed by two-way ANOVA. Day: *F*_5,168_ = 10.45, *P* < 0.0001; Neu3: *F*_1,168_ = 0.9944, *P* = 0.3201. **c** The swimming speed of the mice was documented during the probe trial and analyzed using Mann-Whitney test, *P* = 0.2358. **d** Analysis of the maximum time spent on the target island was conducted using Student’s *t* test. *P* = 0.3716. **e** The total distance traveled by the mice in each quadrant was recorded and analyzed through two-way ANOVA. Quadrant: *F*_3,112_ = 4.041, *P* = 0.0091; Neu3: *F*_1,112_ = 0.4175, *P* = 0.5195. EGFP, n = 16; Neu3, n = 14. **f** Experimental design flowchart. At 6 months of age, 3xTg-AD female underwent unilateral hippocampal injection of either AAV-EGFP or AAV-Neu3. The Morris water maze test was conducted at 8 months of age. **g** Experimental design flowchart. At 4 months of age, male PS19 or littermate control subjects (Con) underwent unilateral hippocampal injection of either AAV-EGFP or AAV-Neu3. The Morris water maze test was conducted at 6 months of age. **h** During the training phase, the latency of mice in **f** was analyzed by two-way ANOVA. Day: *F*_3,40_ = 1.305, *P* = 0.2861; Neu3: *F*_1,40_ = 0.2748, *P* = 0.6030. EGFP, n = 5; Neu3, n = 7. **i** The swimming speed of mice in **f** during the probe trial was analyzed using Student’s *t* test, *P* = 0.8874. **j** The latency of mice in **f** to reach the escape platform was analyzed by Mann-Whitney test, *P* = 0.8674. **k** The distance traveled in each quadrant of mice in **f** was analyzed by two-way ANOVA. Quadrant: *F*_3,40_ = 1.322, *P* = 0.2807; Neu3: *F*_1,40_ = 0.0491, *P* = 0.8258. **l** During the training phase, the latency of mice in **g** was analyzed by two-way ANOVA followed by uncorrected Fisher's LSD test. Day: *F*_3,76_ = 0.8421, *P* = 0.4750; Genotype: *F*_2,76_ = 7.283, *P* = 0.0013. Con vs. PS19+EGFP, *P* = 0.0008; Con vs. PS19+Neu3, *P* = 0.0055; PS19+EGFP vs. PS19+Neu3, *P* = 0.8644. Con, n = 9; PS19+EGFP, n = 8; PS19+Neu3, n = 5. **m** The swimming speed of mice in **g** during the probe trial was analyzed using one-way ANOVA, *P* = 0.8584. **n** The latency of mice in **g** to reach the escape platform was analyzed by Kruskal-Wallis test, *P* = 0.1160. **o** The distance traveled in each quadrant of mice in **g** was analyzed by two-way ANOVA. Quadrant: *F*_3,76_ = 10.71, *P* < 0.0001; Genotype: *F*_2,76_ = 0.1560, *P* = 0.8558. ­

**Supplementary Figure 3 Neu3 reduced oligomeric tau levels in AD P-tau-injected 3xTg-AD mice, without altering spatial learning or Aβ plaque intensity. a** The latency during the training days of the Morris water maze test of EGFP+P-tau and Neu3+P-tau mice (Fig. 2a) was recorded and analyzed using **t**wo-way ANOVA. Day: *F*_3,64_ = 19.15, *P* < 0.0001; Neu3: *F*_1,64_ = 0.4086, *P* = 0.5249. **b** The swimming speed of the mice in water maze was recorded and analyzed **t**wo-way ANOVA **followed by** **uncorrected Fisher's LSD post hoc test**. Quadrant: *F*_3,64_ = 1.580, *P* = 0.2030; Neu3: *F*_1,64_ = 12.20, *P* = 0.0009. EGFP+P-tau, n = 10; Neu3+P-tau, n = 8. **c** The density of Aβ plaques in the hippocampus of each group was quantified and analyzed with **t**wo-way ANOVA. Side: *F*_1,36_ = 3.476, *P* = 0.0704; Neu3: *F*_3,36_ = 1.704, *P* = 0.1836. EGFP+saline, n = 4; Neu3+saline, n = 4；EGFP+P-tau, n = 6；Neu3+P-tau, n = 8. **d** Mice brains underwent immunofluorescence using tau oligomers antibody (T22) and AT8. Hoechst stains the nuclei. **e** The intensity of T22 in mice hippocampus was quantified (n =3) and analyzed with **t**wo-way ANOVA. Side: *F*_1,16_ = 15.77, *P* = 0.0011; group: *F*_3,16_ = 44.48, *P* < 0.0001; Side × group interaction: *F*_3,16_ = 7.743, *P* = 0.0020.

**Supplementary Figure 4 The effect of gangliosides on AD P-tau-seeded tau aggregation and mice behavior. a-b** HEK293T cells were treated with varying concentrations of GM1 for 24 h. Following treatment, cells were lysed, and dot blot analysis was conducted to detect GM1 and GD1a levels. **a** Quantification of GM1 intensity was analyzed using one-way ANOVA (n =4), *F* = 3.710, *P* = 0.0175.**b** Quantification of GD1a intensity was analyzed using one-way ANOVA (n =4), *F* = 2.628, *P* = 0.0593. **c-d** HEK293T cells were transfected with pCI/HA-tau_151-391_, induced with AD P-tau for tau aggregation, and treated with 80 μM GM1 for 42 h. The RIPA insoluble and soluble fractions were assessed via Western blot using anti-HA antibodies. GAPDH in RIPA-soluble fractions served as a loading control. **d** The ratio of insoluble to soluble tau in **c** was quantified (n = 3) and analyzed by two-way ANOVA. P-tau: *F*_1,8_ = 142.1, *P* < 0.0001; GM1: *F*_1,8_ = 4.458, *P* = 0.0677; P-tau × GM1: *F*_1,8_ = 9.178, *P* = 0.0163. **e-f** HEK293T cells were treated with 80 μM GD1a for 24 h. After lysis, dot blot analysis was performed to measure GM1 and GD1a levels. **e** Quantification of GM1 intensity was analyzed using **Student’s *t* test (**n = 3**),** *P* = **0.2515**. **f** Quantification of GD1a intensity was analyzed using **Student’s *t* test (**n = 3**). g-h** HEK293T cells were transfected with pCI/HA-tau_151-391_, induced with AD P-tau for tau aggregation, and treated with 80 μM GD1a for 42 h. The RIPA insoluble and soluble fractions from cell lysates were isolated and subjected to Western blot using anti-HA antibodies. GAPDH in RIPA-soluble fractions served as a loading control. **h** The ratio of insoluble to soluble tau in **g** was quantified (n = 3). **T**wo-way ANOVA, P-tau: *F*_1,8_ = 50.39, *P* = 0.0001; GD1a: *F*_1,8_ = 0.0655, *P* = 0.8045. **g-i** 20-month-old wild-type mice were unilaterally injected with AD P-tau into the hippocampus. After 2 weeks, the mice received intraperitoneal GM1 administration at a dose of 50 mg/kg for 5 consecutive days, monthly over a duration of 5 months, followed by behavioral testing. **i** The mean speed of the mice in the open field was recorded and analyzed using Kruskal-Wallis test, *P* = 0.7130. Con, n = 5; P-tau+saline, n = 9; P-tau+GM1, n= 6. **j** The total number of arm enteries in the Y-maze was recorded and analyzed using one-way ANOVA, *F* = 2.278, *P* = 0.1329. Con, n = 5; P-tau+saline, n = 9; P-tau+GM1, n= 6. **k** The distance traveled by the mice in each quadrant of the water maze was measured and analyzed through two-way ANOVA. Quadrant: *F*_3,63_ = 2.386, *P* = 0.0772; treatment: *F*_2,64_ = 1.018, *P* = 0.3671. Con, n = 5; P-tau+saline, n = 8; P-tau+GM1, n= 6.

**Supplementary Figure 5 Neu3 modulates the GD1a/GM1 ratio in SH**-**SY5Y cells, while GD1a-mediated TF555 uptake is dependent on LRP1. a-c** SH-SY5Y cells were transfected with pcDNA3.1/Neu3 and analyzed with immunofluorescence using anti-Neu3 and anti-GD1a antibodies. Hoechst stained the nuclei. **b** Quantification of GD1a intensity in **a** was analyzed using **Student’s *t* test (**n = 3**), *P* = 0.1311. c** Quantification of GM1 intensity was analyzed using **Student’s *t* test (**n = 3**)** (figure in Fig. 5d)**. d** The GD1a/GM1 ratio was calculated and analyzed using **Student’s *t* test (**n = 3**). e-h** SH-SY5Y cells were transfected with siNeu3 and immunostained with anti-GD1a and anti-GM1 antibodies. **f** Quantification of GD1a intensities was analyzed using **Student’s *t* test (**n = 3**), *P* = 0.4224. g** Quantification of GM1 intensities was analyzed using **Welch's *t* test (**n = 3**). h The GD1a/GM1 ratio was calculated and** analyzed using **Student’s *t* test (**n = 3**). i-j** SH-SY5Y cells were transfected with siLRP1 and immunostained with anti-LRP1 antibodies. **j** Quantification of LRP1 intensities was analyzed using **Student’s *t* test (**n = 3**). k-l** SH-SY5Y cells were treated with 80 μM GD1a, 5 µg/mL anti-LRP1 antibody, or a combination of GD1a and LRP1, followed by exposure to TF555 (figures for Con and GD1a treatment were not shown). **l** Quantification of TF555 intensity was analyzed by two-way ANOVA**.** GD1a: *F*_1,12_ = 4.595, *P* = 0.0532; anti-LRP1: *F*_1,12_ = 23.03, *P* = 0.0004; GD1a × anti-iLRP1: *F*_1,12_ = 2.880, *P* = 0.1154 (n = 4).

**Supplementary Figure 6 Docking interaction of AD PHF with monosaccharides components of GD1a. a Docking interaction of PHF (purple) with galactose (blue). b Docking interaction of PHF with GalNAc (blue). c, Docking interaction of PHF with glucose (blue). Contacting residues are** illustrated **in stick representation (green). Yellow dashed lines, hydrogen bonds.**

**Supplementary Materials and Methods**

*Immunofluorescence and lectin staining*

Cells or frozen sections of brain tissue were washed three times with phosphate buffered saline (PBS) and fixed with 4% paraformaldehyde (BL539A, Biosharp Life Sciences, Hefei, China) for 10 min. Following fixation, the samples were treated with 0.3% Triton X-100 in PBS for 15 min at room temperature. Subsequently, the samples were blocked with a solution containing 5% normal goat serum, 0.1% Triton X-100, and 0.05% Tween 20 in PBS for 30 min. The slices were then incubated overnight at 4°C with primary antibodies diluted at 1:500 (as detailed in Supplementary Table 1). After incubation, the primary antibody was washed out with PBS, and the slices were further incubated with secondary antibodies at a dilution of 1:500 for 2 h at room temperature. The secondary antibodies used included Alexa Fluor® 488 AffiniPure™ goat anti-rabbit IgG (111-545-144), Alexa Fluor® 488 AffiniPure™ goat anti-mouse IgG (115-545-062), Cy™3-AffiniPure™ goat anti-rabbit IgG (111-165-144), Cy™3 AffiniPure™ goat anti-mouse IgG (115-165-146), and Cy™5-AffiniPure™ goat anti-mouse IgG (115-175-146), all obtained form Jackson ImmunoResearch Inc., West Grove, PA, USA. To label the nuclei, the samples were stained with 1 μg/mL Hoechst 34580 (Thermo Fisher Scientific). The sections were coverslipped using ProLong™ Gold Antifade Reagent (P36930, Thermo Fisher Scientific).

**Supplementary Table 1. Antibodies used in this study**.

| **Antibody** | **Type** | **Species** | **Source (catalog No.)** |
| --- | --- | --- | --- |
| Aβ (4G8) | Mono | Mouse | BioLegend (800701, San Diego, CA, USA) |
| AT8 (pS202/T205 tau) | Mono | Mouse | Thermo Fisher Scientific (MN1020) |
| Flag | Poly | Rabbit | Proteintech (20543-1-AP, Rosemont, IL, USA) |
| Flag | Mono | Mouse | Abmart (M20008S, Shanghai, China) |
| GAPDH | Poly | Rabbit | Bioss (bs-41373R, Beijing, China) |
| GD1a | Mono | Mouse | MilliporeSigma (MAB5606Z) |
| GM1 | Poly | Rabbit | Abcam (ab23943, Cambridge, UK) |
| HA | Poly | Rabbit | Proteintech (51064-2-AP) |
| LRP1 | Poly | Rabbit | Bioss (bs-10920R) |
| Neu3 | Poly | Rabbit | Proteintech (27879-1-AP) |
| Tau | Poly | Rabbit | Proteintech (10274-1-AP) |
| Tau (HT7) | Mono | Mouse | Invitrogen (MN1000) |
| Tau oligomer (T22) | Poly | Rabbit | MilliporeSigma (ABN454-I) |
| TAU5 | Mono | Mouse | Millipore (MAB361/1816394) |

For lectin staining, after fixation, the slices were incubated with 5 μg/mL Cy™3-conjugated SNA (CL-1303-1, Vector Laboratories, Newark, CA, USA) for 4 h at room temperature. Alternatively, slices were incubated with 5 μg/mL biotinylated MAL II (B-1265-1, Vector Laboratories) for 2 h, following by incubation with 10 μg/mL Dylight™ 549 Streptavidin (SA-5549-1, Vector Laboratories) for an additional 2 h at room temperature.

Imaging was performed using a Leica THUNDER Imager Tissue microscope (Leica Microsystems, Wetzlar Germany), a ZEISS Axio Imager M2 epifluorescence microscope, or a LSM900 confocal microscope (Carl Zeiss Microscopy, Jena, Germany). Images were processed using Adobe Photoshop v11.0.2 and Adobe Illustrator CS6 v.16.0.0 (Adobe Systems Incorporated, San Jose, CA). Fluorescence intensity was quantified using Adobe Photoshop. The average number of AT8+ neurons in the hippocampus was quantified from 6 brain sections per mouse.

*Behavioral tests*

The animals were transported to the testing room 5 days prior to the experiment and acclimated for 5 min per day. All behavioral tests were conducted between 8 AM and 5 PM, and data were recorded using ANYmaze video tracking system (Stoelting Co., Wood Dale, IL, USA). The apparatus were thoroughly cleaned with water and 75% ethanol, and subsequently dried with paper towels between each trial.

For open field, the mice were placed in a 50 cm × 50 cm × 40 cm arena and allowed to explore for 10 min. A central zone measuring 18 cm ×18 cm was defined at the center of the apparatus, where the mice were initially introduced. The duration of time spent by the mice in the central zone was recorded and analyzed.

For the Y-maze task, a Y-shaped arena with three arms of identical dimensions (35 cm in length, 8 cm in width, and 15 cm in height) arranged at 120° angles was utilized. Each subject mouse was placed in one arm and allowed to explore the Y-maze for 5 min. A consecutive entry into all three arms was classified as a spontaneous alternation. The percentage of the spontaneous alternation rate was calculated by dividing the number of spontaneous alternations by the total number of entries into the three arms, reflecting the mice's ability to recognize new environments and their short-term spatial memory [1].

The Morris water maze consisted of a circular pool measuring 180 cm in diameter and 60 cm in height, filled with nontoxic white dye-tinted water and maintained at room temperature. Animals unable to swim in the water maze were excluded from the final analysis. The maze was theoretically divided into four equal quadrants by two perpendicular lines. A platform, submerged 1 cm below the water surface, was positioned centrally in one quadrant and remained fixed throughout all trials. Each mouse underwent 4 trials per day for 5 consecutive days, starting from semi-randomly assigned positions, with the goal of locating the hidden platform within a 90 s time limit. Upon finding the platform, the mouse was allowed to remain on it for 20 s. If a mouse failed to locate the platform within the allotted time, it was gently guided to the platform and permitted to stay for 20 s. After each trail, the mouse was dried and returned to its home cage. A probe test was performed 24 h after the final trail, during which the platform was removed, and the mouse was allowed to swim in the maze for 90 s.

*Western blots, dot blots and lectin blots*

The hippocampus was homogenized in a 9-fold volume of ice-cold lysis buffer comprising 50 mM Tris-HCl (pH 7.4), 8.5% sucrose, 2 mM EDTA, 10 mM β-mercaptoethanol, 1 mM Na_3_VO_4_, 50 mM NaF, and a 10% protease inhibitor cocktail. The homogenates or cell lysate were then mixed with an appropriate volume of 5 × Laemmli sample buffer (P0015, Beyotime, Shanghai, China) and boiled for 10 min. Protein concentration was determined using the Pierce™ 660 nm Protein Assay kit (22660, Thermo Fisher Scientific). The samples were subjected to 10% sodium dodecyl sulfate-polyacrylamide gel electrophoresis (SDS-PAGE) and subsequently transferred onto a polyvinylidene fluoride (PVDF) membrane (MilliporeSigma).

For dot blots, cells or brain tissue were lysed in Tris-buffered saline (TBS) with a 10% protease inhibitor cocktail. The samples were then spotted onto a nitrocellulose membrane (Schleicher and Schuell, Keene, NH, USA) at 3 to 5 μL per grid within a 7 × 7 mm area. The blot was incubated at 37°C for 1 h to facilitate protein binding to the membrane.

The membranes were first blocked with 5% fat-free milk in TBS for 30 min and then incubated overnight with specific primary antibodies (see Supplementary Table 1). After incubation, the membrane was washed with TBST (0.05% Tween-20 in TBS) and exposed to horseradish peroxidase (HRP)-conjugated secondary antibodies (Jackson ImmunoResearch Inc.) for 2 h at room temperature.

For lectin blots, the PVDF membrane containing the protein sample was blocked with protein-free T20 (TBS) blocking buffer (#3751, Thermo Fisher Scientific) for 1 h. Following this, the membrane was incubated at room temperature with either 1 μg/mL biotin-conjugated SNA or 2 μg/mL biotin-conjugated MAL II for 30 min. The membrane was subsequently washed three times with PBS containing 0.3% Tween-20 to remove unbound lectins. Finally, the membrane was incubated with PBS containing 1 μg/mL HRP-conjungated streptavidin (SA-5014-1, Vector Laboratories) for an additional 30 min.

After antibody incubation, the membranes were treated with Immobilon® ECL UltraPlus Western HRP Substrate (WBULP, MilliporeSigma) and detected using the Tanon 5200 Chemiluminescence Image Analysis System (Tanon, Shanghai, China). The intensity of bands on Western blots and lectin blots was quantified using MultiGauge software v3.0 (Fuji Film, Minato, Tokyo, Japan). The intensity of dot blots was measured with ImageJ 1.48v (National Institutes of Health, Bethesda, MD, USA).

*Liquid chromatography coupled to tandem mass spectrometry (LC-MS/MS)*

The LC-MS/MS analysis was performed by Applied Protein Technology (Shanghai, China). Briefly, lipids were extracted using the methyl-tert-butyl ether (MTBE) method. Mouse hippocampus was mixed with 200 μL of water and 20 μL of an internal lipid standard mixture, followed by the addition of 800 μL of MTBE. The mixture was then combined with 240 μL of pre-cooled methanol. The samples were sonicated for 20 min and left at room temperature for 30 min. Subsequently, the samples were centrifuged at 14000 × g at 10 °C for 15 min. The upper organic phase was collected and dried under nitrogen. The lipid extracts were re-dissolved in 200 μL of a 90% isopropanol/acetonitrile solution, centrifuged at 10 °C for 15 min at 14000 × g, and the supernatant was used for LC-MS/MS. After separation using a UHPLC Nexera LC-30A (SHIMADZU, Kyoto, Japan), the samples were analyzed with a Q-Exactive Plus mass spectrometer™ (Thermo Fisher Scientific).

*Molecular docking*

Molecular docking was performed by Autodock (La Jolla, CA, USA). The lowest free energy cluster returned by AutoDock for each compound was present in the text.

*Statistical analysis*

Quantitative data are presented as mean ± standard error of the mean (SEM). The exact sample size of each experiment is provided in the relevant figure legend. The sample size was chosen based on common practices in the field for similar types of experiments, as reported in previous literature [2-5]. The data were initially assessed for distribution using the Shapiro-Wilk test. For two groups that conform to a normal distribution (*P* > 0.05 in the Shapiro-Wilk test), an F-test is used to assess the homogeneity of variances. If the *P*-value from the F-test is less than 0.05, Welch's *t* test is applied; otherwise, a two-tail Student's *t* test is utilized. For comparisons involving three or more groups of normally distributed data, the Brown-Forsythe test is employed to assess variances, followed by one-way ANOVA and an uncorrected Fisher's **least significant difference** (LSD) post hoc test. In cases where data were not normally distributed (*P* < 0.05 in the Shapiro-Wilk test), the Mann-Whitney test was used for two-group comparisons, while the Kruskal-Wallis test followed by uncorrected Fisher's LSD post hoc test was applied for three or more groups. Other datasets were analyzed using two-way ANOVA followed by the uncorrected Fisher's LSD post hoc test. All statistical analyses were conducted using GraphPad Prism 9.0.2 (GraphPad Software Inc., San Diego, CA, USA). *P* < 0.05 was considered statistically significant.

**References**

1.	Jung MJ, Kim N, Jeon SH, Gee MS, Kim JW, Lee JK. Eugenol relieves the pathological manifestations of Alzheimer's disease in 5xFAD mice. Phytomedicine 2023; **118:** 154930.

2.	Guo JL, Lee VM. Seeding of normal Tau by pathological Tau conformers drives pathogenesis of Alzheimer-like tangles. J Biol Chem 2011; **286**(17)**:** 15317-15331.

3.	Holmes BB, DeVos SL, Kfoury N, Li M, Jacks R, Yanamandra K *et al.* Heparan sulfate proteoglycans mediate internalization and propagation of specific proteopathic seeds. Proc Natl Acad Sci U S A 2013; **110**(33)**:** E3138-3147.

4.	Li L, Miao J, Chu D, Jin N, Tung YC, Dai CL *et al.* Tau antibody 77G7 targeting microtubule binding domain suppresses proteopathic tau to seed tau aggregation. CNS Neurosci Ther 2022; **28**(12)**:** 2245-2259.

5.	Dai CL, Hu W, Tung YC, Liu F, Gong CX, Iqbal K. Tau passive immunization blocks seeding and spread of Alzheimer hyperphosphorylated Tau-induced pathology in 3 x Tg-AD mice. Alzheimers Res Ther 2018; **10**(1)**:** 13.
